# The Housing of the Working Classes Act

**Published:** 1897-05-22

**Authors:** 


The Hospital

A Journal of

The Medical Sciences and Hospital Administration.

Vol. XXII.—No. 556.] [May 22, 1897.

### The Housing of the Working Classes Act.

One item in the budget presented to the London County Council last week was on account of workmen's dwellings.

With the economic side of the question of providing comfortable dwellings for the working classes we have no concern at the present moment. Our concern is with the influence of this movement on the public health, and so it may be added was the concern of those by whom the Act was framed, and what we especially wish to insist on is that, to get out of this Act the full sanitary advantage which it is capable of giving to the community, the main parts into which it is divided, viz., Part I. and Part II., should be applied *pari passu*, and always be coupled with a strict enforcement of the sanitary law.

However much one may doubt as to the economic soundness of a policy which tends to give to certain selected people what is undoubtedly eleemosynary aid, in the form of lowered rent or better houses for the same rent, and however much we may question the ultimate effect of such aid upon the wages of the district in which it is given, no one can look at the houses which are being put up by the London County Council and compare them with the tenements which they replace without confessing that, whatever economists may say to the performance, there is a sanitary gain to the districts in question.

The problem, however, does not end here. We have further to consider the effect of these clearances upon other districts and upon other houses in the same district, and one cannot but see that, notwithstanding the careful provisions of the Act in regard to rehousing, such rehousing is absolutely impossible in many instances, and that, as the tendency of hungry people always is, and always must be, to economise in rent rather than in bread, those who are turned out must be driven downwards in the scale of comfort, unless some countervailing influence be brought to bear. Of course we are aware that the new buildings profess to be let at the rent ruling in the districts, but it must be obvious to anyone that the tendency of erecting healthy, and even handsome, buildings is to raise the general character of a district and make it an acceptable residence for people of a higher class, and thus to raise the criterion by which the rents are fixed. Moreover, even if the rents keep down, the superior accommodation offered draws tenants from other classes, and thus it inevitably happens that Part I. of the Act, standing by itself, must often tend to sink into still deeper depths the lot of the residuum.

Fortunately the Public Health (London) Act, 1891, and Part II. of the Housing of the Working Classes Act provide a means of preventing such evil consequences, and there can be no doubt that by slow degrees, and by the continuous exercise of the powers thus given to the local authorities, they could, if they chose, raise the standard of dwelling and the standard of comfort below which no one in this great city should be allowed to descend. This is the object which should be aimed at; not the mere erection of a few model dwellings. These are but a means to an end.

The difficulties in the way of harmonious action are, however, considerable, owing to the fact that the duty of putting these various provisions into operation lies in the hands of different bodies, and that, while the County Council can deal on a general plan with large unhealthy areas, and can cover them with workmen's dwellings, that gradual driving upwards of the standard of sanitation in the whole metropolis, on which depends the sanitary utility of these dwellings, lies with vestries and local bodies, who by no means always enter into this work with enthusiasm and never on any general plan. Hence the great advantage and necessity of the local authorities being controlled and supervised by such a central body as the London County Council. Recent events in Marylebone have shown how backward local authorities may be in raising the standard of living, and how necessary some such form of supervision as is now in operation may be. During the last five or six years the County Council has been engaged in making a systematic inspection, more especially of the central and poorer parts of London, and have constantly sought by publication of what has been done in the several districts to raise the general standard of sanitary administration in the metropolis. We find that the following districts have been thus inspected: Whitechapel, Mile End Old Town, Holborn, Clerkenwell, Bethnal Green, Lambeth, Fulham, and Rotherhithe, and that, in view of the fact that overcrowding still remains the great difficulty, the County Council, after inquiry as to the extent to which the various local authorities are undertaking the regulation of houses let in lodgings, have formally resolved that any metropolitan authority which after Midsummer of this year does not definitely proceed to exercise its powers in this matter shall be reported to the Local Government Board as making default in the administration of the Public Health Act.

This is as it should be. Unless there is a general raising of the standard all round one parish becomes the dumping ground for the residuum of the others, and the erection of a few workmen's dwellings is a mere placebo.

